# French Amyloidosis CAPS study: AA Amyloidosis complicating cryopyrin-associated periodic syndrome: a study on 14 cases and review of 53 cases from literature

**DOI:** 10.1186/1546-0096-13-S1-P32

**Published:** 2015-09-28

**Authors:** S Georgin-Lavialle, K Stankovic Stojanovic, D Buob, P Quartier, B Neven, I Kone-Paut, E Hachulla, A Belot, L Cuisset, S Anselm, G Grateau

**Affiliations:** 1 AP-HP Tenon hospital, Internal Medicin, Paris, France; 2 AP-HP- Necker hospital, pediatric rheumatology, Paris, France; 3 AP-HP Kremlin-Bicêtre hospital, pediatric rheumatology, Kremlin-Bicêtre, France; 4 CH Lyon Sud, Rheumatologic pediatry, Lyon, France; 5 CHU Lille, Internal Medicin, Lille, France; 6 AP-HP Cochin hospital, Genetics, Paris, France; 7 AP-HP Trousseau hospital, Genetics, Paris, France

## Background

The cryopyrin-associated periodic syndrome (CAPS) is a rare but treatable inherited autoinflammatory condition including familial cold autoinflammatory syndrome (FCAS), Muckle-Wells syndrome (MWS) and chronic infantile neurologic cutaneous articular syndrome (CINCA). Without treatment, some patients develop AA amyloidosis with consequent renal failure and death.

## Objective

To describe the main features of CAPS-associated AA amyloidosis and the efficacy of interleukin-1 inhibitors in this complication.

## Methods

We retrospectively analysed all current French CAPS-associated amyloidosis cases through the French network for rare diseases, and performed a systematic literature review of such cases published since 1950.

## Results

Fourteen French patients were identified (6 women/8 men) including MWS (n=9), FCAS (n=3), CINCA (n=2) and having received interleukin-1 inhibitors in 7 cases. Mean age at the diagnosis of amyloidosis was 22.6 years and five (35.7 %) patients died. We found 53 patients in the literature, with a sex ratio of 1. They included MWS (n=34), FCAS/MWS (n=12) and FCAS (n=7). Among 67 patients (French and literature), the median age at amyloidosis diagnosis was 30 years, ranging from 12 to 61 years. The *NLRP3* gene was sequenced in 30 patients (45.5%), and the distribution of amino acids changes was as follows: R262W (n=16), T348M (n=5), A439V (n=4), D303N (n=3), T436N (n=2) and L353P (n=1). 23 patients had died (35%), but none of them had received interleukin-1 inhibitors. Since 2002, 24/67 (36%) patients with CAPS-associated amyloidosis have received interleukin-1 inhibitors, with at least a decrease of proteinuria and creatininemia in 9 of them (37.5%)

## Discussion

AA amyloidosis can occur in all CAPS phenotypes, even if it was more frequent in MWS. This study underlines that even if FCAS is considered as a milder clinical phenotype compared to MWS or CINCA, it can also lead to amyloidosis. Thus, if FCAS patients display continuous subclinical inflammation, they should receive Interleukin-1 inhibitors as well, in order to prevent AA amyloidosis. Interleukin-1 inhibitors were introduced since a few years (anakinra and canakinumab), and it is still unclear if they can cure secondary amyloidosis. However, in 39% of cases they could allow a decrease of both proteinuria and creatininemia. In addition, in our experience and in the literature, anti IL1 treatments were able to prevent amyloidosis-related fatality.

## Conclusion

AA amyloidosis can occur in all types of CAPS. IL-1 inhibitors prevent the occurrence of AA amyloidosis and should be started as soon as possible, even in FCAS patients in case of subclinical inflammation.

